# Myocardial injury after major non-cardiac surgery evaluated with advanced cardiac imaging: a pilot study

**DOI:** 10.1186/s12872-023-03065-6

**Published:** 2023-02-10

**Authors:** Jesús Álvarez-Garcia, Ekaterine Popova, Miquel Vives-Borrás, Miriam de Nadal, Jordi Ordonez-Llanos, Mercedes Rivas-Lasarte, Abdel-Hakim Moustafa, Eduard Solé-González, Pilar Paniagua-Iglesias, Xavier Garcia-Moll, David Viladés-Medel, Rubén Leta-Petracca, Gerard Oristrell, Javier Zamora, Ignacio Ferreira-González, Pablo Alonso-Coello, Francesc Carreras-Costa

**Affiliations:** 1 Department of Cardiology, Hospital Universitario Ramon y Cajal, M-607, 9,100, 28034 Madrid, Spain; 2 Department of Cardiology, Hospital de la Santa Creu i Sant Pau, Sant Quintí 89, 08026 Barcelona, Spain; 3 Centro de Investigación Biomédica en Red Enfermedades Cardiovaculares (CIBERCV), Madrid, Spain; 4 IIB SANT PAU, Institut d’Investigació Biomèdica Sant Pau, Sant Quintí 77, 08041 Barcelona, Spain; 5 Centro Cochrane Iberoamericano, Sant Antoni Maria Claret, 167, 08025 Barcelona, Spain; 6 Fundació Institut d’Investigació Sanitària Illes Balears (IdISBa), Department of Cardiology, Carretera de Valldemossa, 79, 07120 Palma, Balearic Islands Spain; 7 Department of Anaesthesiology and Intensive Care, Hospital Universitari Vall d’Hebron, Passeig de la Vall d’Hebron, 119, 08035 Barcelona, Spain; 8 Department of Biochemistry, Hospital de la Santa Creu i Sant Pau, Sant Quintí 89, 08026 Barcelona, Spain; 9 Foundation for Clinical Biochemistry & Molecular Pathology, Barcelona, Spain; 10 Department of Cardiology, Hospital Universitario Puerta de Hierro Majadahonda, C. Joaquín Rodrigo, 1, 28222 Majadahonda, Madrid, Spain; 11 Department of Cardiology, Hospital Clinic i Provincial, C. de Villarroel, 170, 08036 Barcelona, Spain; 12 Department of Anaesthesia and Pain Management, Hospital de la Santa Creu i Sant Pau, Sant Quintí 89, 08026 Barcelona, Spain; 13 Department of Cardiology, Hospital Universitari Vall d’Hebron, Passeig de la Vall d’Hebron, 119, 08035 Barcelona, Spain; 14 Clinical Biostatistics Unit, IRYCIS, Hospital Universitario Ramon y Cajal, M-607, 9,100, 28034 Madrid, Spain; 15 CIBER Epidemiología y Salud Pública (CIBERESP), Madrid, Spain; 16 Department of Cardiology, Hospital Universitari Son Espases, Carretera de Valldemossa, 79, Palma, Illes Balears Spain

**Keywords:** Myocardial injury, Noncardiac surgery, Cardiac imaging, Pathophysiology

## Abstract

**Background:**

Myocardial injury after non-cardiac surgery (MINS) is a frequent complication caused by cardiac and non-cardiac pathophysiological mechanisms, but often it is subclinical. MINS is associated with increased morbidity and mortality, justifying the need to its diagnose and the investigation of their causes for its potential prevention.

**Methods:**

Prospective, observational, pilot study, aiming to detect MINS, its relationship with silent coronary artery disease and its effect on future adverse outcomes in patients undergoing major non-cardiac surgery and without postoperative signs or symptoms of myocardial ischemia. MINS was defined by a high-sensitive cardiac troponin T (hs-cTnT) concentration > 14 ng/L at 48–72 h after surgery and exceeding by 50% the preoperative value; controls were the operated patients without MINS. Within 1-month after discharge, cardiac computed tomography angiography (CCTA) and magnetic resonance imaging (MRI) studies were performed in MINS and control subjects. Significant coronary artery disease (CAD) was defined by a CAD-RADS category ≥ 3. The primary outcomes were prevalence of CAD among MINS and controls and incidence of major cardiovascular events (MACE) at 1-year after surgery. Secondary outcomes were the incidence of individual MACE components and mortality.

**Results:**

We included 52 MINS and 12 controls. The small number of included patients could be attributed to the study design complexity and the dates of later follow-ups (amid COVID-19 waves). Significant CAD by CCTA was equally found in 20 MINS and controls (30% vs 33%, respectively). Ischemic patterns (n = 5) and ischemic segments (n = 2) depicted by cardiac MRI were only observed in patients with MINS. One-year MACE were also only observed in MINS patients (15.4%).

**Conclusion:**

This study with advanced imaging methods found a similar CAD frequency in MINS and control patients, but that cardiac ischemic findings by MRI and worse prognosis were only observed in MINS patients. Our results, obtained in a pilot study, suggest the need of further, extended studies that screened systematically MINS and evaluated its relationship with cardiac ischemia and poor outcomes.

*Trial registration* Clinicaltrials.gov identifier: NCT03438448 (19/02/2018).

**Supplementary Information:**

The online version contains supplementary material available at 10.1186/s12872-023-03065-6.

## Introduction

Annually, over 300 million people undergo major noncardiac surgery worldwide [1]. Despite preoperative screening, surgical improvements and increased patient monitoring, myocardial infarction remains the first cardiovascular cause of morbidity and mortality within 30 days after surgery [2]. Atherothrombosis is the underlying cause for most non-operative myocardial infarctions; but the mechanisms of the myocardial injury in noncardiac surgery (MINS), including perioperative myocardial infarction, are multiple and difficult to identify in the usual clinical practice. Theoretically, myocardial injury may be caused by four distinct mechanisms: coronary plaque rupture [3, 4], myocardial oxygen supply–demand mismatch [5, 6], non-ischemic cardiac disorders, such an atrial fibrillation episode [7], or non-cardiac causes, such as pulmonary embolism [8]. However, the angiographic, histological, or imaging studies required to identify all the MINS etiological mechanisms of are difficult to be implemented in all patients undergoing non-cardiac surgery [9]. Better understanding of causes originating MINS could help to develop potential preventive and therapeutic interventions. Recently, cardiac computed tomography angiography (CCTA), has demonstrated to improve the value of revised cardiac risk index [10], an established prognostic indicator of major cardiac events after surgery [11]. Moreover, cardiac magnetic resonance imaging (MRI) is considered the gold standard for noninvasively study of myocardial functionality. Therefore, minimally invasive diagnostic tests, like CCTA and cardiac MRI, could be promising tools to identify the occurrence and elucidate the underlying mechanisms of MINS.

In this pilot study, we aimed to identify with CCTA and MRI the existence of non-clinically evident coronary artery disease (CAD) and/or focal myocardial fibrosis in patients with or without MINS after undergoing major non-cardiac surgery. In addition, we aimed to analyse the relationship between MINS occurrence and the future major cardiovascular events (MACE). The primary outcomes were prevalence of CAD among MINS and controls and incidence of MACE at 1-year. Secondary outcomes were incidence of all 1-year outcomes, including mortality and individual components of MACE.

## Methods

We adhered to the STROBE reporting guidelines (Additional file 1: Table S1). The protocol and the informed consent for troponin sampling and cardiac image studies were approved by the Ethics Committee of Clinical Research of the Hospital de la Santa Creu i Sant Pau in May 11th, 2016. The other participating centre (Hospital Vall d´Hebron) adhered to this approval (permitted by our local legislation) and used the same informed consents. The study was registered at Clinicaltrials.gov (NCT03438448). All participants provided written informed consent before recruitment.

### Study design

The current was a prospective, observational, cohort study in patients undergoing major noncardiac (elective or urgent) surgery (mainly digestive, gynaecologic, neurosurgery, orthopaedic, otorhinolaryngologic, plastic, thoracic, traumatological and vascular), requiring at least an overnight hospital admission. The study was carried out in two University Spanish hospitals, between July 2016 and December 2019. In the one of the participating centres (Hospital de la Santa Creu i Sant Pau) the current study was one of the sub-studies of a large cohort study aiming to evaluate the feasibility and impact of implementation of the systematic preoperative and postoperative hs-cTnT screening, as well as its cost-effectiveness., where systematic hs-cTnT screening program for MINS, was implemented at the routine perioperative care. Therefore, all patients provided informed consent for troponin sampling before surgery. In the other centre (Hospital Vall d´Hebron), where hs-cTnT screening was not performed systematically, the samplings were included in the framework of the study using the same informed consent for troponin sampling before surgery. All included patients at the cardiac imaging study, from two participating centres were identified and invited to participate after surgery and provided their specific imaging study informed consent.

### Study participants

The study was focused on acute postoperative myocardial injury, i.e., MINS (excluding myocardial infarction). All patients had to meet at least one of the following inclusion criteria: (1) age ≥ 65 years old, (2) antecedents of stroke or transient ischemic attack or peripheral vascular disease if < 65 years old, or (3) preoperative estimated glomerular filtration rate (eGFR) between 30–59 mL/min/1.73 m^2^ Exclusion criteria included: (1) antecedents of ischemic heart disease and/or chronic heart failure, and (2) any contraindication to perform cardiac CCTA or MRI. Patients fulfilling inclusion criteria were identified by research personnel at post-surgery units, invited to participate in the image study and those accepting to be included signed a specific informed consent.

### Hs-cTnT measurements

In the included patients, we measured the high-sensitive cardiac Troponin T (hs-cTnT, Roche Diagnostics, Basel, Switzerland) at three times: preoperatively and 48 and 72 h after surgery. The values of the limit of detection, 99th upper reference percentile (URL) and 10% coefficient of variation were 5.0 ng/L, 14.0 ng/L (both sexes) and 13.0 ng/L, respectively.

### MINS definition and management

When a postoperative rise and/or fall pattern in hs-cTnT, with at least one value above the URL, was detected in a patient a 12-lead electrocardiogram (ECG) was performed. If the postoperative ECG showed changes vs the ECG before surgery, an echocardiography was conducted to rule out wall motion abnormalities. After completing the process, MINS was defined as any postoperative hs-cTnT value higher than the URL and showing at least a 50% increase respect to the preoperative concentration, in a patient without ECG signs or symptoms of myocardial ischemia. The control group included the patients without hs-cTnT elevations. A structured cardiology consultation was performed in MINS and control groups.

### Advanced cardiac imaging studies (CCTA and cardiac MRI)

CCTA and MRI were performed in all patients within the first month after discharge and at the same centre (Hospital de Sant Pau, who acted as core-lab for cardiac imaging). On the previous days of the CCTA, patients were treated with a beta-blocker (atenolol 25–50 mg or ivabradine 5–7.5 mg to achieve a heart rate ≤ 60 beats per minute). A pair of expert evaluators formed by a cardiologist and a radiologist with level 3 training in interpretation of CCTA, read each angiogram using a 17-segment model of the coronary arteries without knowledge of the clinical data. Per-patient anatomical severity was classified according to the Coronary Artery Disease—Reporting and Data System (CAD-RADS) [12]. Triple-rule-out CCTA examinations (coronary artery disease, pulmonary embolism, and acute aortic pathology) were also performed. After the CCTA study, an MRI exam was performed to evaluate the global and segmental cardiac contractility and presence of focal fibrosis, using late gadolinium enhancement (LGE) contrast. We classified the LGE pattern as “ischemic” if subendocardial or transmural delayed contrast enhancement in a vascular distribution was observed and “non-ischemic” if enhancement was distributed patchy or diffuse, not following a vascular territory, mainly in mesocardial or epicardial locations [13]. Finally, in those with significant CAD in the CCTA (CAD-RADS ≥ 3), a pharmacological stress with adenosine was conducted, to assess functional impact of each coronary stenosis. A more detailed version of the study protocol was previously published [14].

### Follow-up and data collection

All patients were followed for the study outcomes at 1 month, and at 1 year, after the date of surgery. The follow-up visits were conducted by telephone, supported by clinical electronic records. If the patients (or relatives) indicated that they had experienced any of the main outcomes, we obtained the relevant source documents from the corresponding electronic health records. All variables including risk factors (including the Revised Cardiac Risk Index-RCRI- [15]), comorbidities, medical treatment, and perioperative data (intraoperative hypotension was defined as a 30% drop of systolic blood pressure from baseline, intra and postoperative bleeding were defined as a 30 g/L drop from preoperative haemoglobin, need for transfusion or requiring haemostatic surgery, and intra and postoperative shock) were collected by study personnel on case report forms and entered at secure online database (www.clinapsis.com).

### Main outcomes

The primary outcomes were prevalence of CAD among MINS and no-MINS patients and incidence of MACE at 1-year. MACE was defined as a composite of myocardial infarction, unstable angina, need of cardiac revascularization, heart failure, new atrial fibrillation episode, stroke, or pulmonary embolism (according definitions of the most recent guidelines). All-cause death was also registered. Secondary outcomes were incidence of all 1-year outcomes, including mortality and individual components of MACE.

### Statistical considerations

#### Sample size

In the original protocol of this cardiac imaging sub-study, it was estimated that it would be necessary to recruit 260 participants (130 MINS cases and 130 matched controls) to detect an association between MINS condition and significant coronary atherosclerosis.

To calculate these sample sizes, we assume a prevalence of significant coronary atherosclerosis of around 19% in our high-risk population. The prevalence data was based in a previous study [16] of some of the current co-authors in a similar population to that of the study.

#### Statistical analysis

For categorical variables, the percentage and the number of cases and the mean and standard deviation or median and interquartile range for quantitative variables were provided. Comparisons between groups were assessed with the Student’s T test or the Mann–Whitney’s U-test for continuous variables and with the chi-square tests or the Fisher exact test for comparing the proportions of categorical variables. Two-sided significance levels of 0.05 were used in all analyses. Data were analysed using STATA SE Version 13.0 (StataCorp LLC, College Station, TX, USA).

## Results

### Clinical characteristics of the study population and risk assessment of index surgery

From total 373 screened MINS patients, 58 did not fulfilled inclusion criteria, other 192 declined to participate, additional 5 withdrawn from the study after had provided informed consent, and finally, 66 were not included due to logistic issues (discharged before complete sampling, CCTA not available, frailty). Therefore, we included 52 MINS patients and completed their follow-up. Regarding the control group and owing to the large number of eligible patients (1,972), we proposed to participate to a similar number no-MINS patients as the needed to achieve our previous size calculations. Unfortunately, in this group we obtained even lower participation rate than in MINS, mainly due to the same reasons as in the MINS group plus a huge number of refusals. We could only include and complete the follow-up in 12 controls. In the recruiting centre with a systematic MINS screening a 10.5% of screened patients were lost by lack on hs-cTnT value at 72 h after surgery. The 1-year follow-up of several cases coincide with the first and second COVID-19 waves in Spain; thus, an indeterminate number of missed follow-ups, particularly in control subjects (no-MINS), could be attributed to the inability of some elderly, frail patients to answer to the follow-up request due to their current clinical condition. Most patients (58, 90.6%) were ≥ 65 years old (mean age 75.1), 30 (46.9%) were females, all Caucasians, with a high prevalence of cardiovascular risk factors mainly hypertension, diabetes mellitus and different dyslipidaemias, and, accordingly, on current treatment with antihypertensive drugs, statins, and aspirin. Peripheral artery disease and chronic obstructive pulmonary disease were also frequent (~ 15%). There were no relevant differences in the baseline features of patients between MINS and control groups, though diabetes mellitus was found in 42.3% (22) of MINS patients and in 16.7% (2) of controls (Table 1).

**Table 1:** Preoperative clinical characteristics of the study population

	Total (64)	Patients with MINS (52; 81.2%)	Controls* (12; 18.8%)	*P* value
Age, years	75.1 [9.3]	75.2 [9.9]	74.5 [6.2]	0.762
≥ 65 years	58 (90.6)	47 (90.4)	11 (91.7)	0.891
Female sex	30 (46.9)	24 (46.2)	6 (50.0)	0.810
Body mass index (kg/m^2^)	22.3 [3.5]	22.0 [3.7]	23.5 [2.5]	0.109
Stroke	5 (7.8)	4 (7.7)	1 (8.3)	0.941
Transient ischemic attack	1 (1.6)	1 (1.9)	0 (0)	1.000
Peripheral artery disease	9 (14.1)	8 (15.4)	1 (8.3)	1.000
Hypertension	45 (70.3)	37 (71.2)	8 (66.7)	0.759
Diabetes mellitus	24 (37.5)	22 (42.3)	2 (16.7)	0.184
Dyslipidaemia	33 (51.6)	25 (48.1)	8 (66.7)	0.245
COPD	10 (15.6)	9 (17.3)	1 (8.3)	0.672
Chronic kidney disease	8 (12.5)	8 (15.4)	0 (0)	1.000
Deep vein thrombosis	1 (1.6)	0 (0)	1 (8.3)	1.000
**Treatments**				
ASA	21 (33.3)	19 (36.5)	2 (16.6)	0.310
Statins	29 (46.0)	23 (44.2)	6 (50.0)	0.533
Oral anticoagulants	4 (6.4)	3 (5.8)	1 (8.3)	0.546
Beta-blockers	4 (6.4)	3 (5.8)	1 (8.3)	0.546
ACEI	35 (55.6)	30 (57.7)	5 (41.7)	0.458

Data are expressed as: number (%), mean [standard deviation], or median (interquartile range) value, as appropriate

MINS: myocardial injury in non-cardiac surgery; COPD: chronic obstructive pulmonary disease; ASA: acetylsalicylic acid; ACEI: angiotensin-converter enzyme inhibitors

*Controls = patients without MINS

Regarding risk assessment prior to index surgery, the RCRI class I was separately analysed from the other classes, since it was the most frequently observed among the patients, to try to avoid that a difference on risk assessment between MINS and controls could remain «unseen» in the general statistical assessment of the fourth RCRI groups. Low RCRI was more frequent in controls (11, 91.7%) than in MINS patients (31, 59.6%, *p* < 0.01); eGFR was lower, in patients with MINS than in controls (71.1 vs. 76.5 mL/min/1.73 m^2^) though the difference was not statistically significant (*p* = 0.136) (Table 2). Hs-cTnT concentrations were not different between MINS and controls previously to surgery, but the MINS group showed median values two to three times higher than controls at 48 h (41 vs 14 ng/L, *p* < 0.002) and 72 h (31 vs 11 ng/L, *p* < 0.001) of surgery. During intervention, hypotension was frequent in MINS and controls (65.3% and 83.3%, respectively) requiring therapy in most cases (71.0% and 90.0%, respectively). In the MINS group, intraoperative bleeding (10.4%) and intra and postoperative shock (16% and 10%, respectively) were found compared with their complete absence in the control group, though owing the few cases the differences between groups did not reach statistical significance.

**Table 2:** Values of the Revised Cardiac Risk Index (RCRI) of index surgery and peri and postoperative complications in patients with MINS and controls

	MINS (52; 81.2%)	Controls (12; 18.8%)	*P* value
**RCRI**			
I	31 (59.6)	11 (91.7)	0.01
II	15 (28.9)	1 (8.3)	1.000
III	2 (9.6)	0 (0)	1.000
IV	1 (1.9)	0 (0)	1.000
**Preoperative BP (mm Hg)**			
Systolic	134 (124–146)	138 (119–159)	0.727
Diastolic	71 (60–83)	79 (70–86)	0.207
**Preoperative HR (bpm)**	74 (63–80)	70 (64–76)	0.534
**Preoperative haemoglobin (g/L)**			
≤ 100	3 (5.8)	1 (9.0)	0.670
101–129	28 (53.9)	5 (45.5)	
≥ 130	21 (40.3)	5 (45.5)	
**eGFR (mL/min/1.73 m**^**2**^**)**	71.1 [16.7]	76.5 [9.0]	0.136
**Hs-cTnT (ng/L)**			
Preoperative	11 (9–16)	11 (8–12)	0.284
48 h post-surgery	41 (23–73)	14 (11–32)	0.002
72 h post-surgery	31 (18–48)	11 (10–13)	0.001
**Priority of surgery**			
Elective	43 (82.7)	8 (66.7)	0.243
Urgent	9 (17.3)	4 (33.3)	
**Type of surgery**			
Orthopaedic	22 (42.3)	8 (66.7)	
General (Digestive)	17 (32.7)	2 (16.7)	
Vascular	7 (13.5)	1 (8.3)	
Neurosurgery	1 (1.9)	1 (8.3)	
Thoracic	4 (7.7)	0 (0)	
Others	1 (1.9)	0 (0)	0.451
**Intraoperative hypotension**	32 (65.3)	10 (83.3)	0.309
Requiring treatment**	22 (71.0)	9 (90.0)	0.402
**Bleeding**			
Intraoperative	5 (10.4)	0 (0)	1.000
Postoperative	9 (18.0)	2 (18.2)	1.000
**Shock**			
Intraoperative	8 (16.0)	0 (0)	1.000
Postoperative	5 (10.0)	0 (0)	1.000
**Ischemic symptoms or signs like ECG**	3 (5.8)	0 (0)	1.000

Data are expressed as: number (%), mean [standard deviation], or median (interquartile range) value, as appropriate

MINS: myocardial injury in non-cardiac surgery; Controls: patients without MINS; RCRI: revised cardiac risk index; BP: blood pressure; HR: heart rate; bpm: beats per minute; eGFR: estimated glomerular filtration rate according to CKD-EPI equation; mL: millilitre; min: minute; m: meter; hs-cTnT: high-sensitive cardiac troponin T; ng: nanogram; L: litter; h: hour

*Controls = Patients without MINS

**Inotropic or vasopressor therapy required

### Advanced cardiac imaging findings

Out of 64 patients, we performed an echocardiogram, a CCTA scan, and a cardiac MRI in 64 (100%), 62 (97%), and 49 (77%), respectively. Table 3 describes the main findings of the advanced cardiac imaging studies. The median left ventricular ejection fraction (LVEF) measured by echocardiogram was normal in both groups, and abnormal wall motion was observed only in three MINS patients. The frequency of significant CAD (CAD-RADS ≥ 3) was similar in MINS (n = 15, 30%) than in the control group (n = 4, 33%); a total of nineteen subjects of both groups had a CAD-RADS ≥ 3. Calcified plaques were found in approximately two thirds of patients of both groups (29 MINS: 58.0%, 7 controls: 58.3%); vulnerable plaques were quite infrequent. In the MRI explorations, LVEF and abnormal wall motion findings were similar than those of CCTA. Late gadolinium enhancement (LGE) was similarly found in near to one third of MINS (n = 11, 28.2%) and controls (n = 3, 30.3%). An adenosine test was performed in the nineteen subjects with a significant CAD by the CAD-RADS index. No differences were observed between groups in the frequency of abnormalities of wall perfusion, as it was found in the echocardiographic or MRI explorations; we found cardiac ischemic patterns in five and ischemic segments in two MINS patients compared with any in the control group.

**Table 3:** Findings in advanced cardiac imaging in patients with MINS and controls

	MINS	Controls	*P* value
**Echocardiography (n = 62)**	**52 (100%)**	**12 (100%)**	**–**
LVEF, %	65 (60–65)	63 (60–65)	0.733
Abnormal wall motion	3 (5.8)	0 (0)	1.000
**CCTA (n = 62)**	**50 (96.2%)**	**12 (100%)**	**–**
Agatston score	143 (0–600)	128 (0–583)	0.889
CAD-RADS 0	11 (22.0)	1 (8.3)	0.833
CAD-RADS 1–2	24 (48.0)	7 (58.3)	
CAD-RADS 3	10 (20.0)	3 (25.0)	
CAD-RADS 4–5	5 (10.0)	1 (8.3)	
Calcified plaque	29 (58.0)	7 (58.3)	0.983
Vulnerable plaque	2 (4.0)	1 (8.3)	0.482
**Cardiac MRI (n = 49)**	**39 (75.0)**	**10 (83.3)**	**–**
LVEF, %	63 (7)	64 (4)	0.563
Abnormal wall motion	1 (2.6)	1 (10.0)	0.370
LGE positive	11 (28.2)	3 (30)	1.000
*Ischemic pattern*	5 (45.5)	0 (0)	1.000
Microvascular obstruction	2 (5.1)	0 (0)	1.000
Abnormal wall perfusion**	4 (26.7)	1 (25.0)	1.000
Ischemic segments**	2 [0–5]	0 [0–0]	1.000
Mod-severe PIQS score**	1 (0–1)	0 (0–0)	1.000

Bold values indicate number of subjects in each group

Data are expressed as: number (%), mean [standard deviation], or median (interquartile range) value, as appropriate

MINS: myocardial injury in non-cardiac surgery; LVEF: left ventricular ejection fraction; CCTA: cardiac computed tomography angiography; CAD: coronary artery disease; MRI: magnetic resonance imaging; LGE: late gadolinium enhancement; PIQS: perfusion index quantitative score

*Controls = patients without MINS

**Adenosine test performed only in those patients with CAD-RADS (Coronary Artery Disease-Reporting and Data System) ≥ 3 (N = 19)

### Outcomes in the follow-up

All cause-mortality (4 patients, 7.7% after 1-year follow-up) only occurred in the MINS group; a sudden cardiac death happened in a patient with many treated cardiovascular risk factors and the other deaths were due to malignancies. MACE was also only detected in MINS patients (n = 8, 15.4%) (Table 4). Three patients with MINS developed ischemic symptoms; two of them undergone coronary angiography, which showed significant coronary artery disease. Both patients were revascularized during the procedure without further complications. The third patient with ischemic symptoms was managed with medical treatment, because due to his/her fragility a coronary interventionism was discouraged.

**Table 4:** One-year outcomes in patients with MINS and controls

	MINS (52; 81.2%)	Controls (12; 18.8%)
All-cause mortality	4 (7.7)	0
MACE	8 (15.4)	0
Unstable angina	3 (5.8)	0
Myocardial infarction	0	0
Cardiac revascularization	2 (3.9)	0
Heart failure	0	0
New atrial fibrillation	2 (3.9)	0
Stroke	1 (1.9)	0
Pulmonary embolism	0	0

Data are expressed as number (%), mean (standard deviation), or median (interquartile range), as appropriate

MINS: myocardial injury in non-cardiac surgery; MACE: major adverse cardiovascular events

*Controls = Patients without MINS

## Discussion

In the current study, conducted in a group of patients with high cardiovascular risk undergoing major non-cardiac surgery, we have analysed the frequency of myocardial injury (MINS) detected with high-sensitive troponin T (hs-cTnT), the occurrence of subclinical coronary artery disease (CAD) detected with advanced imaging techniques, and the occurrence of cardiovascular complications after one year follow-up.

We found several interesting findings. First, the Revised Cardiac Risk Index (RCRI) stage I, associated to the lowest cardiac risk, was significantly more frequent in control subjects than in MINS patients. Though differences in the preoperative variables included in the RCRI between both groups did not achieve statistical significance, MINS patients received more often thoracic, abdominal, and vascular surgeries and have an eGFR lower than controls; taking together, all these differences could justify the predominance of a RCRI value associated to low cardiac risk in the control population. Second, in our study, we measured hs-cTnT pre and postoperatively and MINS was defined by percentual increases against the preoperative concentration. In the MINS group, the postsurgical hs-cTnT concentrations were only mildly elevated (2–3 times) over the upper reference limit (URL) of 14 ng/L, but between 3 to 4-times over the preoperative values. This result support that the extent of myocardial injury in the MINS group was small and that it was easier to detect by hs-cTnT serial changes rather than by reference to the URL. Our findings agree with the low individuality index of hs-cTnT observed in different studies both in control subjects and in patients with cardiac or renal disease [17]. When the individuality index of a variable is low, the signification of its value must be analysed against its serial evolution rather than against its URL. Moreover, patients as those included in our study often have basal hs-cTnT values higher than the URL; thus, to define an ongoing myocardial injury serial values and significant changes are required [18]. Third, by CCTA we found that significant CAD, measured as a CAD-RADS ≥ 3.0, existed in 30% of MINS and in 33.3% of controls; these findings superseded the assumptions made in our protocol. Our results agreed with those of OPTIMUS [19] and CORONARY Vision-CTA [11] studies, which observed that atherothrombosis was implicated in one third of cases of cardiac damage in non-cardiac surgery patients. However, as outlined in a sub-study of the CORONARY Vision-CTA study, many patients with severe coronary lesions in the CCTA did not have postoperative complications suggesting that CCTA could overestimate the future risk of these patients. The authors of the study suggested that MRI could improve the identification of patients at risk of postoperative outcomes [20]. Of note, the CCTA explorations in the CORONARY Vision-CTA were performed preoperatively and, in our study, postoperatively. Fourth, by late gadolinium enhanced cardiac MRI, we identified focal fibrosis, a sign of myocardial ischemia, in one-third of both MINS patients and controls. Our population, although exclude patients with previous ischemic cardiac disease or chronic heart failure, included many subjects with cardiovascular risk factors or antecedents of cerebrovascular and peripheral arterial diseases. Thus, our patients would be prone to have subclinical myocardial ischemic features in MRI; same MRI findings have been observed in large studies in asymptomatic individuals or general population presenting similar health status than our patients [21, 22],. There are only one study using MRI to detect postoperative myocardial injury after non-cardiac surgery in 22 patients with an age and health status similar to ours and with significative CAD detected by CCTA, although myocardial injury was assigned using a contemporary cardiac troponin I assay with lower analytical and clinical sensitivity than high sensitive assays used in our study [23]. The study found clinically silent pulmonary embolism in one-third of patients with myocardial injury, an alteration that we did not observe in our study. Late gadolinium enhancement and perfusion defect were observed in one-third of cases, same proportion as in our study. The small number of included patients in the referred study and in own study could be the cause of highly discrepant proportions, but really derived from very small numbers. Fifth, adenosine stimulation, only conducted in patients with a CAD-RADS ≥ 3.0, revealed that unlike the similar frequency of the index and abnormal wall reperfusion defects in both MINS and controls, only MINS group had ischemic segments in MRI. Pharmacological stress perfusion with adenosine is the non-invasive investigation of choice in patients with suspected, but uncertain myocardial ischemia diagnosis [24]. As mentioned, our patients, both those with MINS and those without, have an increased preoperatory risk of cardiovascular complications. However, despite the similarities between both groups in some cardiac postoperative image features, the adenosine stimulation revealed that ischemic segments only existed in MINS patients. Thus, adenosine stimulation was a useful tool to finely distinguish patients with non-clinically evident cardiac lesions that are associated with poorer prognosis as observed in our MACE results. Sixth, intraoperative hypotension requiring therapy was equally frequent in MINS and controls; however, intraoperative bleeding and intra and postoperative shock were only found in MINS. Both the bleeding and shock occurring in the MINS patients are well-known causes of myocardial oxygen supply–demand mismatch. Thus, bleeding and shock, linked with a frequent although undetected CAD, are probably the ultimate causes of many of the observed MINS in our study. These results agreed with those found in the OPTIMUS and CORONARY Vision-CCTA studies which attributed two-thirds of the myocardial damage observed after non-cardiac surgery to the supply–demand imbalance mechanism, whereas only one-third could be attributed to atherothrombosis [11, 19]. And, seven, whatever the cause at their origin, the occurrence of MINS was associated with a 15% frequency of MACE in the 1-year follow-up, whereas in the control group any MACE was detected in the follow-up. This result outlines the importance of MINS screening and diagnosis.

### Implications for practice and research

Understanding the pathophysiology of MINS is crucial to develop potential prophylactic and therapeutic interventions to improve the prognosis of patients undergoing noncardiac surgery. Our results, as hypothesis generating from a pilot study, must be confirmed by further large studies, but could inform prophylactic and therapeutic interventions, and eventually, improve the prognosis of MINS patients. Moreover, our study was performed in two Spanish hospitals, so further large research involving a more racially diverse study population will help to extend the clinical implications of our results.

### Limitations and strengths

We acknowledge several limitations of our study. First, we were unable to achieve initially estimated sample size. A small size, with a low number of patients in the control group, makes difficult to provide some statistically significant conclusions. As mentioned, we screened 373 individuals in each group, but the losses due to failing a complete hs-cTnT sampling, the reluctancy of the controls to undergone imaging tests, the difficulty for displacements of old, frail patients recently operated and the temporal coexistence of part of the 1-year follow-up with COVID waves that restricted the participation of individuals could explain our small recruitment. A study with a very close design as our protocol, included 46 patients with myocardial injury and 20 controls after screening 1205 candidates in pre-COVID times [23]. Therefore, our results should be interpreted as a pilot study, providing a “proof of concept” paving the way to design further studies in this field. Second, the assumptions made at the time of initial protocol development were superseded by the higher significant CAD-RADS ≥ 3 atherosclerosis frequency found by CCTA both in MINS (30.0%) and controls without MINS (33.3%) (Table 3). Third, we assessed coronary anatomy by CCTA and cardiac functionality by MRI after surgery; thus, it is possible that some of the cardiac findings existed before the intervention. Fourth, our selection criteria resulted in a population of patients with not known CAD and with or without isolated MINS; our results may be difficult to apply to other populations.

Regarding strengths, our study has some. The study addresses a very important topic in perioperative medicine, often not addressed in clinical practice, and provides some new knowledge using fine techniques as high-sensitive cardiac troponin, CCTA and cardiac MRI. In our knowledge, this is the first application of CCTA together with cardiac MRI in the evaluation of major non-cardiac surgery patients. We identified two different groups in our subjects with some similarities in coronary anatomy and occurrence of coronary disease features, but clear differences regarding cardiac ischemia and adverse outcomes in the follow-up that only were observed in patients with MINS. These observations reinforce the need of implementing systematic screening in major non-cardiac surgery patients to identify MINS and implement therapies that could decrease their occurrence.

## Conclusions

This study with advanced imaging methods found a similar CAD frequency in MINS and control patients, but that cardiac ischemic findings in the MRI exploration and worse prognosis were only observed in MINS patients. Our results, obtained in a pilot study, suggest the need of further, extended studies that screened systematically MINS and evaluated its relationship with cardiac ischemia and poor outcomes.

## Supplementary Information

**Additional file 1.** STROBE-Checklist.

## Data Availability

The data used in the present study is part of a larger dataset. The datasets generated and analysed during the current study are available and can be supplied from the corresponding authors upon reasonable request. The data not used for this manuscript will be employed in future manuscripts.

## Abbreviations

MINS: Myocardial injury after non cardiac surgery
CAD: Coronary artery disease
CAD-RADS: Coronary Artery Disease-Reporting and Data System
CCTA: Cardiac computed tomography angiography
MRI: Magnetic resonance imaging
ECG: Electrocardiogram
MACE: Major cardiovascular events
hs-cTnT: High-sensitive cardiac troponin
URL: Upper reference percentile
RCRI: Revised Cardiac Risk Index
LGE: Late gadolinium enhancement
LVEF: Left ventricular ejection fraction
